# Changing the Electron Acceptor Specificity of *Rhodobacter capsulatus* Formate Dehydrogenase from NAD^+^ to NADP^+^

**DOI:** 10.3390/ijms242216067

**Published:** 2023-11-08

**Authors:** Hemant Kumar, Silke Leimkühler

**Affiliations:** Department of Molecular Enzymology, Institute of Biochemistry and Biology, University of Potsdam, Karl-Liebknecht-Str. 24-25, 14476 Potsdam, Germany; hemant.kumar@uni-potsdam.de

**Keywords:** molybdoenzymes, changing cofactor specificity, formate dehyrogenases, enzyme engineering

## Abstract

Formate dehydrogenases catalyze the reversible oxidation of formate to carbon dioxide. These enzymes play an important role in CO_2_ reduction and serve as nicotinamide cofactor recycling enzymes. More recently, the CO_2_-reducing activity of formate dehydrogenases, especially metal-containing formate dehydrogenases, has been further explored for efficient atmospheric CO_2_ capture. Here, we investigate the nicotinamide binding site of formate dehydrogenase from *Rhodobacter capsulatus* for its specificity toward NAD^+^ vs. NADP^+^ reduction. Starting from the NAD^+^-specific wild-type *Rc*FDH, key residues were exchanged to enable NADP^+^ binding on the basis of the NAD^+^-bound cryo-EM structure (PDB-ID: 6TG9). It has been observed that the lysine at position 157 (Lys^157^) in the β-subunit of the enzyme is essential for the binding of NAD^+^. RcFDH variants that had Glu^259^ exchanged for either a positively charged or uncharged amino acid had additional activity with NADP^+^. The FdsB^L279R^ and FdsB^K276A^ variants also showed activity with NADP^+^. Kinetic parameters for all the variants were determined and tested for activity in CO_2_ reduction. The variants were able to reduce CO_2_ using NADPH as an electron donor in a coupled assay with phosphite dehydrogenase (PTDH), which regenerates NADPH. This makes the enzyme suitable for applications where it can be coupled with other enzymes that use NADPH.

## 1. Introduction

Overall, carbon dioxide (CO_2_) accumulation in the atmosphere is a result of increasing global warming and climate change, and it is one major concern for the world [1]. An increasing amount of research concerning the efficient removal of atmospheric CO_2_ has been carried out in the last few decades. Among the enzymatic methods of CO_2_ removal, formate dehydrogenases (FDHs) have been investigated as CO_2_ reductases, reducing CO_2_ to formate. Compared to conventional methods in the chemical industry, enzymatic methods have the advantage of catalyzing reactions under ambient conditions. Further, enzymes are highly specific compared to their chemical counterparts. Formate can serve as a fuel to be used in fuel cells as a feedstock for chemoautotrophic growth and an energy storage compound for a hydrogen economy [2].

FDHs are an important class of enzymes that preferentially catalyze the oxidation of formate to CO_2_ [3]. They have also been shown to catalyze the CO_2_ reduction reaction; however, formate oxidation is the thermodynamically favored reaction.

$${CO}_{2}+2e^{-}+H^{+}\overset{}{↔}{HCOO}^{-},{E{}^{\circ}}^{\prime }=-420\;mV$$

They are divided into two structurally and catalytically different classes based on their metal dependency [4]. Metal-independent FDHs are found in eukaryotes [5] as well as prokaryotes [6]; in contrast, metal-dependent enzymes are exclusively found in prokaryotes. The former has been well studied with many of the available crystal structures (e.g., FDHs from *Candida boidini* and *Pseudomonas* sp.101). The hydride transfer reaction mechanism of metal-independent FDHs [7] is well accepted, and previous enzyme engineering studies have also led to similar conclusions [8,9]. Moreover, they are frequently used for the regeneration of cofactors in coupled enzymatic assays [10,11,12,13]. However, the major drawbacks of most FDHs are their cofactor preference for NAD^+^ over NADP^+^ and their low activity in the reaction of CO_2_ reduction.

Metal-dependent FDHs, on the other hand, belong to the DMSO reductase family of mononuclear molybdenum or tungsten-containing enzymes. DMSO reductases are among the three families of molybdoenzymes, the other two families being the xanthine oxidase and sulfite oxidase families [14]. Members of the DMSO reductase family contain bis-molybdopterin guanine dinucleotide (bis-MGD) cofactors in their active sites. The active site of metal-containing FDHs comprises one molybdenum or tungsten atom, which is hexacoordinated by two dithiolenes contributed from two molybdopterin guanine dinucleotide (MGD) moieties, a sulfido ligand, and a catalytic cysteine or selenocysteine ligand. They were shown to catalyze the reaction of CO_2_ reduction with higher efficiency compared to their metal-independent counterparts [15].

So far, mainly metal-independent formate dehydrogenases have been used as cofactor (NAD(P)H) recycling systems in enzymatic biocatalytic reactions [16]. They have advantages over other recycling enzymes, mainly because the product CO_2_ is in gaseous form and can easily be separated from the product. This also keeps the equilibrium of the reaction always toward formate oxidation. There have been several examples of protein engineering in order to change the nicotinamide (NAD(P)H) specificity of formate dehydrogenases. FDHs from *C. boidinii, C. methylica,* and *Pseudomonas* sp. are examples where the NAD^+^ binding site has been engineered to use NADP^+^ instead. Engineering of the nicotinamide binding site in formate dehydrogenases has been exclusively performed in metal-independent FDHs so far. This is mainly because these FDHs have been relatively well studied, they are easy to handle, and they are tolerant of oxygen. By comparison, relatively few examples of NAD(P)^+^ serving as an electron acceptor exist [17,18] for metal-dependent FDHs since the modular diaphorase subunit is independent from the catalytic bis-MGD active site. Nonetheless, nicotinamide cofactor specificity, aside from the discovery of NAD(P)^+^-reducing FDHs, has not been studied among structurally and mechanistically different metal-dependent FDHs. Changing the specificity of metal-containing FDHs from NAD^+^ to NADP^+^ will expand the choices of coupling partners for efficient CO_2_ reduction, as revealed by applications using metal-independent FDHs [19]. It is known that metal-dependent FDHs have much higher catalytic rates compared to metal-independent FDHs [15], and this is important for using their more efficient CO_2_ reductase activity.

**Figure 1:**
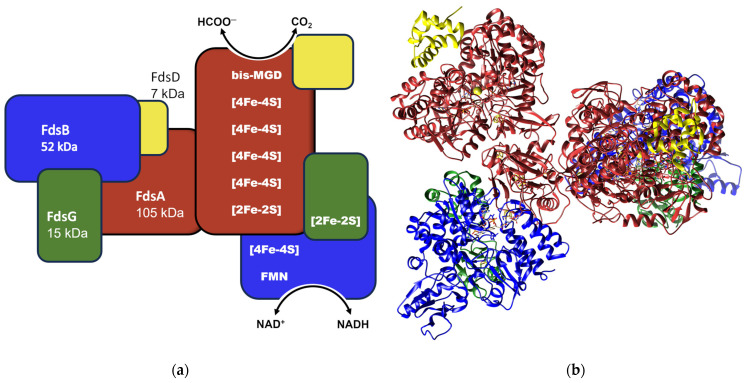
Schematic representation of RcFDH subunits. Subunits are indicated as follows: FdsA, red; FdsB, blue; FdsG, green; FdsD, yellow (**a**). Ribbon representation of the atomic model derived from the cryo-EM structure (PDB-ID: 6TG9) with subunits differentiated by the similar colors as depicted in (**a**). Cofactors are shown in stick presentation (**b**).

Formate dehydrogenase from *Rhodobacter capsulatus* (RcFDH) is a heterodimeric molybdoenzyme and each functional monomer contains four subunits, as shown in Figure 1a,b. The active site containing the FdsA subunit has a bis-MGD cofactor, four [4Fe-4S] clusters, and two [2Fe-2S] clusters. Subunits FdsG and FdsB together form the diaphorase subunit of RcFDH. The FdsG subunit has one [2Fe-2S] cluster, while the FdsB subunit has one [4Fe-4S] and a FMN moiety and constitutes the NAD^+^ binding site. RcFDH has been well studied for its formate oxidation and CO_2_ reduction activities [20,21,22,23,24], in addition to inhibition studies. Due to the availability of the cryo-EM structure and the establishment of a heterologous expression system in *E. coli*, RcFDH is the ideal enzyme to employ site-directed mutagenesis for mechanistic studies [25].

In this study, *Rhodobacter capsulatus* formate dehydrogenase (RcFDH) (Figure 1a,b) was engineered to react with NADPH instead of NADH as a reductant. The wild-type enzyme (RcFDH-WT) initially had no activity toward NADP^+^. Based on a homology model of RcFDH, the putative residues surrounding the NAD^+^ cofactor were targeted for site-directed mutagenesis, and the generated RcFDH variants were expressed and purified heterologously in *E. coli*. These variants were further characterized, and their kinetic parameters were determined for both NAD^+^ and NADP^+^.

## 2. Results

### 2.1. Production of RcFDH Variants at the NAD^+^ Site

**Figure 2:**
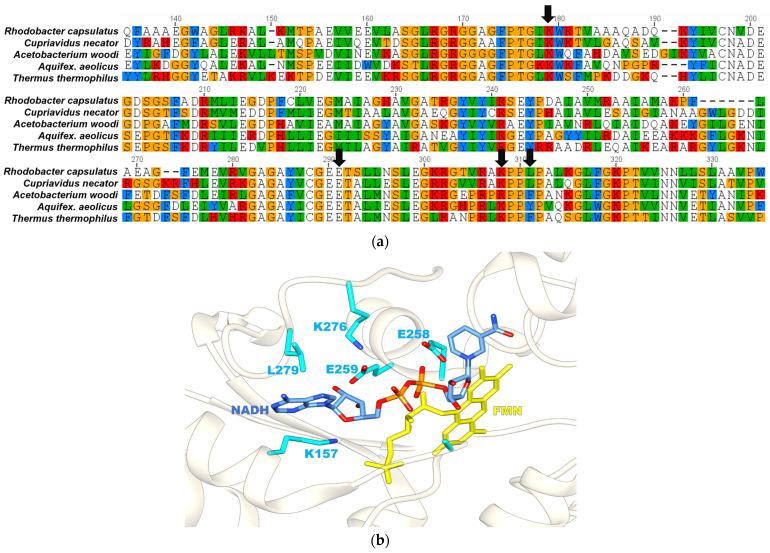
Multiple sequence alignment of FdsB subunit of *Rc*Fdh with diaphorase subunits of other organisms using COBALT from NCBI [30]. Color code for amino acids are as follows: Y,F,W = blue; R,K,H = red; G,S,T,R = yellow; V,L,M,I = green; and D,E,N,Q,A,C = no color. NAD^+^ binding site residues are marked by black arrows (**a**). Cartoon representation of NAD^+^ binding site of RcFdsB (PDB-ID: 6TG9) (**b**).

The RcFDH^WT^ enzyme was subjected to engineering to change the cofactor specificity from NAD^+^ to NADP^+^. A sequence alignment of the FdsB subunit from *R. capsulatus* with the diaphorase subunit of complex I from *Thermus thermophilus* (TtRCI, PDB-ID: 2FUG) [26] showed 36.7% amino acid identity among the two (Figure 2a). It also showed a high amino acid identity of 62% to the chain B of formate dehydrogenase from *Cuprivadus necator* (PDB-ID: 6VW7) [27], 41.1% identity to Fe hydrogenase from *Acetobacterium woodi* (PDB-ID: 7Q4V) [28], and 37.7% identity to the NADH-quinone oxidoreductase subunit F from *Aquifex aeolicus* (pbd id: 6HL2) [29]. Among the residues at the RcFDH NAD^+^ binding site in the cryo-EM structure (PDB-ID: 6TG9), i FdsB^Lys157^ and FdsB^Glu259^ in particular were positioned at a hydrogen-bonding distance from the NAD^+^ cofactor (Figure 2b). The adenosine diphosphate (ADP) moiety of NAD^+^ had a similar orientation to that of TtRCI (PDB-ID: 3IAM), where these Lys and Glu residues formed a hydrogen bond with the nicotinamide cofactor. However, in the case of the RcFDH cryo-EM structure, the nicotinamide ring of NADH/NAD^+^ was facing away from the FMN isoalloxazine ring structure, most likely representing a non-productive binding mode, reflecting the substrate inhibition of NADH at high concentrations [21]. To change the cofactor specificity of *R. capsulatus* FDH from NAD^+^ to NADP^+^, we selected amino acids from the cryo-EM structure of the NADH-bound enzyme that are likely involved in substrate specificity. The four key residues, FdsB^Lys157^, FdsB^Glu259^, FdsB^Lys276^, and FdsB^Leu279^, were chosen.

**Figure 3:**
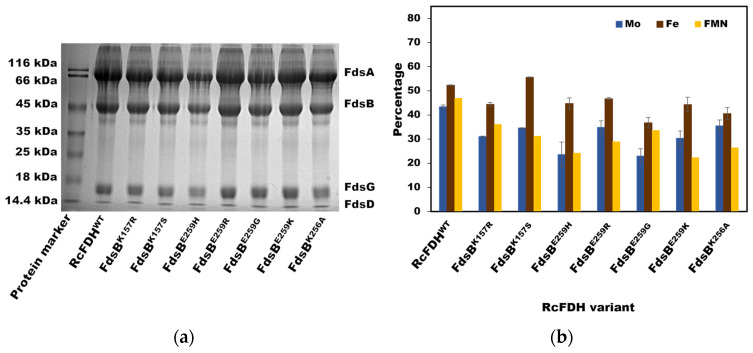
A total of 15% SDS-polyacrylamide gel of purified RcFDH^WT^ and variants after size exclusion chromatography. Samples contained 20 µg of protein (**a**). Molybdenum, iron, and FMN saturations of the RcFDH variants relative to RcFDH^WT^ as quantified by inductively coupled plasma optical emission spectroscopy (ICP-OES) and FMN quantitation, respectively. A total of 1 mol of fully saturated monomeric enzyme contains 1 mol Mo, 24 mol Fe, and 1 mol FMN that was set to a cofactor saturation of 100%, and the measured values of the purified proteins were related to that optimal saturation (**b**).

These residues were also suggested by a web tool for nicotinamide cofactor reversal called CSR-SALAD (http://cheme.che.caltech.edu (accessed on 28 September 2021)) when the NAD^+^-bound cryo-EM structure of RcFDH was used as a query [31]. FdsB^Lys157^ was conserved among organisms, as shown in Figure 2a. Therefore, the FdsB^Lys157^ residue was substituted for arginine (FdsB^K157R^) with a positively charged side chain and serine (FdsB^K157S^) with a polar side chain. Further, FdsB^Glu259^ was exchanged for either a positively charged or uncharged amino acid. FdsB^Lys276^ and FdsB^Leu279^ residues are in close proximity to the nicotinamide cofactor in the RcFDH structure. In order to accommodate the additional phosphate group of NADP^+^, FdsB^Lys276^ was replaced with alanine (FdsB^K276A^), and FdsB^Leu279^ was replaced with arginine (FdsB^L279R^). The amino acid exchanges were introduced by site-directed mutagenesis. The RcFDH variants at the NAD^+^ binding site in the FdsB subunit were expressed and purified as isolated (FdsGBAD)_2_ heterodimers and were compared relative to the RcFDH^WT^ enzyme. Expression and purity levels of all the variants were similar to those of the wild-type enzyme, as shown in Figure 3a. Metal quantitation of these proteins via inductively coupled plasma optical emission spectroscopy (ICP-OES) revealed that both molybdenum and iron saturations of variants were slightly lower than that of the wild-type enzyme (Figure 3b). Similarly, the FMN cofactor content of the variants was found to be less than the wild-type enzyme. However, the FMN content of these variants was found to be enough to exclude an apparent lack of activity due to a lowered FMN content.

### 2.2. Lys^157^ of FdsB Subunit Is Essential for the Binding of the Nicotinamide Cofactor

After purification, we tested the activities of the produced variants using either NAD^+^ or NADP^+^ as electron acceptors. Exchanges at position 157 of the FdsB subunit resulted in inactive proteins since both the FdsB^K157R^ and FdsB ^K157S^ variants had no formate oxidation or CO_2_ reduction activity, as shown in Table 1. The metal analysis using ICP-OES (Figure 3b) revealed that these variants had comparable amounts of molybdenum and iron to RcFDH^WT^, showing that the lack of metal was not the reason for their low activity. Since the enzymes were purified in the presence of sodium azide, we assume that the enzyme remains in its active form, as previously shown [24]. Conclusively, FdsB^Lys157^ plays a crucial role in nicotinamide binding.

### 2.3. Cofactor Specificity of RcFDH Variants Testing Formate Oxidation

The kinetic parameters were determined for all the variants and tested for activity in CO_2_ reduction with NADPH instead of NADH as the reductant. As shown in Table 1, RcFDH^WT^ has no detectable activity when NADP^+^ is used as an electron acceptor. In contrast, FdsB^E259R^, FdsB^E259K^, FdsB^E259G^, and FdsB^E259Q^ variants showed formate oxidation activity when NADP^+^ was used as an electron acceptor. However, variant FdsB^E259D^ behaved like RcFDH^WT^ and showed no activity with NADP^+^. All of these variants at position 259 also showed relatively lower formate oxidation activity with NAD^+^. This shows that the removal of the negative charge at position 259 is crucial for NADP^+^ binding. The FdsB ^K157R^ and FdsB^K157S^ variants were completely inactive, showing the critical role of FdsB^Lys259^ for nicotinamide binding. The FdsB^K276A^ and FdsB^L279R^ variants also showed NADP^+^-dependent formate oxidation activity. Interestingly, these two variants still have low activity with NAD^+^. Kinetic parameters of RcFDH variants showed that single mutations at the NAD^+^ binding subunit resulted in activity with NADP^+^ as a cofactor. In order to improve the *K_m_* value for NADP^+^, these single mutations were combined. FdsB^K276A/E259G^, FdsB ^K276A/E259R^, and FdsB ^K276A/L279R^ were created and expressed. Unfortunately, FdsB^K276A/E259G^ was not expressed, while the other two variants did not show any improvement in their *K*_M_ or *k*_cat_ values (Table 1). Among all the variants, the FdsB^E259G^ variant had the highest catalytic efficiency for formate oxidation using NADP^+^ as an electron acceptor.

### 2.4. CO_2_ Reduction Catalyzed by RcFDH Variants Using NADPH

**Figure 4:**
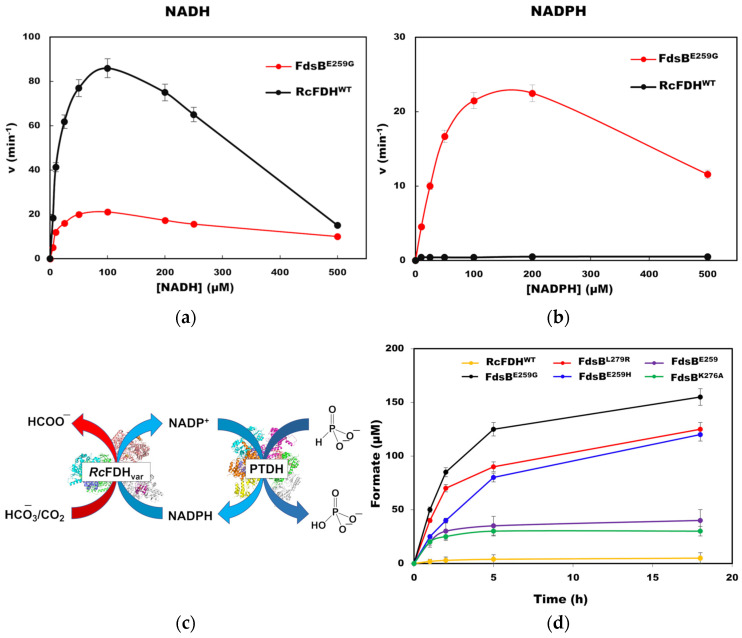
Steady-state kinetics of CO_2_ reduction catalyzed by RcFDH^WT^ and FdsB^E259G^ variants using NADH (**a**) or NADPH (**b**) as the electron donor. CO_2_ reduction is catalyzed by RcFDH variants using NADPH-recycling system with phosphite dehydrogenase. (**c**) Time-dependent formate production by RcFDH^WT^ and variants using NADPH as the electron donor (**d**). A total of 500 µL of reaction volume contained 100 mM of sodium bicarbonate, 100 µM of NADH or NADPH, 10 mM of sodium phosphite, 4 µM of phosphite dehydrogenase (PTDH), 1 µM of FMN, and 50 mM of KP_i_ buffer, pH 6.8. The reaction was started by the addition of 2–10 µM of RcFDH^WT^ or variants, and the vials were incubated for up to 18 h at room temperature. Reactions were stopped by adding 30 µL of acetone to a 100 µL sample, followed by centrifugation at 14,000× *g* for 10 min. The supernatant was collected and derivatized for formate quantification by GC-MS.

Besides formate oxidation, RcFDH^WT^ is also known to catalyze the reverse reaction of CO_2_ reduction to formate when NADH is used as an electron donor [21]. However, at higher concentrations of NADH, substrate inhibition with a *K*_i_ value of 180 µM is observed (Figure 4a). As shown in Figure 4b, RcFDH^WT^ has no CO_2_ reduction activity when NADPH is used as an electron donor. CO_2_ reduction using RcFDH^WT^ and variants was also determined under anaerobic conditions. Since the rate for CO_2_ reduction is about 20× lower than for formate oxidation, the rate was too low to be determined in a time-dependent assay. Therefore, we decided to use a coupled enzyme assay using phosphite dehydrogenase. The recycling of the NADPH was carried out by phosphite dehydrogenase in the reaction mixture, which allowed for the assay to be performed over longer time periods without depleting NADPH in the reaction. Phosphite dehydrogenase reduces NADP^+^ to NADPH by oxidizing phosphite to phosphate (Figure 4a). The reactions were incubated for 18 h, and the product formate was quantified by GC-MS after derivatization. As shown in the kinetic assay data (Table 1), RcFDH^WT^ has negligible activity toward NADPH/NADP^+^ as coenzyme. Correspondingly, RcFDH^WT^ was not able to produce any detectable amounts of formate after 18 h of reaction as per GC-MS measurement in the presence of NADPH (Figure 4d). On the other hand, the RcFDH variants were able to produce readily detectable amounts of formate when NADPH was used instead of NADH. Enzymes had 60% of their activity remaining after 18 h of reaction. Among the variants, FdsB^L279R^ and FdsB^E259G^ showed the highest formate production of up to 120 µM and 150 µM, respectively. The fact that the turnover number of the NADPH cofactor for these two variants is higher than one (1.5 and 1.2, respectively) indicates that the phosphite dehydrogenase-based NADPH-recycling system is functional.

We tested FdsB^E259G^ for CO_2_ reduction kinetics using both NADH and NADPH as electron donors since this variant showed the highest formate oxidation activity using NADP^+^. As shown in Figure 4a,b, FdsB^E259G^ has NADPH-dependent CO_2_ reduction activity with a *k*_cat_ value of 24 ± 4 min^−1^ and a *K*_m_ value of 83 µM. While this variant showed very low inhibition with NADH (Figure 4a), for NADP+, a substrate inhibition (*K*_i_ = 233 µM) was determined (Figure 4b), while FDH^wt^ did not react with NADPH.

## 3. Discussion

Formate dehydrogenases catalyze the reversible reduction of CO_2_ to formate, which can be used, i.e., for the removal of atmospheric CO_2_ to reduce global warming. The molybdoenzyme formate dehydrogenase from *R.capsulatus* (RcFDH) normally uses NADH as the electron donor for CO_2_ reduction [21]. Most of the molybdenum or tungsten-containing formate dehydrogenases, on the other hand, due to differences in their subunit composition, do not react with nicotinamide coenzymes but use other electron mediators [32]. To the best of our knowledge, so far, no investigations have been performed to study the role of NADH-binding residues in determining the coenzyme specificity in the group of molybdenum cofactors containing formate dehydrogenases. However, studies published on diaphorase subunits of complex I and hydrogenase have been performed, and the GB subunit of RcFDH is highly similar to those subunits [33]. Metal-independent formate dehydrogenases, on the other hand, which share no homology to the molybdenum-containing formate dehydrogenases, mainly utilize NAD^+^ as the coenzyme. Metal-independent formate dehydrogenases have been thoroughly investigated regarding the reaction mechanism as well as the cofactor specificity. A recent study on changing the cofactor specificity to NADP^+^ by Ma et al. [34] produced amino acid exchanges predicted by CSR-SALAD at the NAD^+^ binding site. Although they were able to generate NADP^+^-specific variants, the CO_2_-reducing activities of the variants were not analyzed, likely based on their very low activities. Overall, metal-independent formate dehydrogenases are suitable for cofactor recycling applications based on their high stability, but for CO_2_ reduction applications, metal-dependent formate dehyrogenases are the better catalysts.

For the CO_2_ reduction reaction, wild-type metal-containing formate dehydrogenases generally have a much higher turnover number (*k*_cat_) compared to the fastest metal-free formate dehydrogenase, even after extensive enzyme engineering [15]. Hence, exploring metal-containing formate dehydrogenases for CO_2_ reduction reactions is a promising area to study. To expand the choice of a coupling partner, we investigated the NADH binding site of formate dehydrogenase from *Rhodobacter capsulatus* and changed residues in the NAD^+^ binding site to bind NADP^+^. The CO_2_ reduction activity of formate dehydrogenase could be coupled to various cofactor recycling systems, and an additional preference for NADP^+^ would expand the potential coupling partners. For example, Ihara et al. [19] coupled metal-free formate dehydrogenase to photosystem I for light-dependent CO_2_ reduction [19]. Photosystem I, under in vitro conditions, together with ferredoxin and ferredoxin/NADPH reductase, could use light for NADPH production when a sacrificial electron donor is used. Thus, NADPH could be used by an NADPH-dependent formate dehydrogenase for CO_2_ reduction in a cascade reaction with photosystem I. For coupling formate dehydrogenase with potential NADP^+^-specific coupling partners, the NAD+ binding site of formate dehydrogenase was engineered for binding NADPH.

Based on the cyro-EM structure of the NADH binding site of RcFDH (PDB-ID: 6TG9), FdsB residues Lys157, Glu259, Lys276, and Leu279 were identified as the key residues for determining the specificity [25]. Since the NADH in this structure was bound in a catalytically ‘unproductive’ form, a model was also created using the X-ray crystal structure of NAD^+^-bound respiratory complex I from *T. thermophilus* (PDB-ID: 3IAM) as a template [26]. The same residues were identified in close proximity to NADH in both models. These residues were replaced with other amino acids after a rational design of the NADH binding site. The CSR-SALAD tool was also used for the prediction of amino acids involved in substrate specificity [31].

The FdsB^Lys157^ residue seems to play a crucial role in binding the nicotinamide cofactor since variants in this amino acid were completely inactive. The FdsB^Lys157^ residue side chain is connected to the ribose sugar of NADH and the alcohol group of FMN via hydrogen bonds. Changing lysine to serine or to arginine led to a complete loss of activity in both variants. Both variants had other intact cofactors just like the wild-type enzyme, as evident from the molybdenum, iron, and FMN saturations. Overall, the variation of Lys^157^ seems to either lead to no nicotinamide binding or catalytically unproductive binding, resulting in an inactive enzyme.

Further, FdsB^Glu259^, FdsB^Lys276^, and FdsB^Leu279^ residues were analyzed for change in terms of cofactor specificity. The side chain FdsB^Glu259^ is connected to the 2′-hydroxyl of the NADH ribose moiety via a hydrogen bond. Like the residue FdsB^Lys157^, FdsB^Glu259^ is also close to one of the hydroxyl groups of the FMN molecule. Switching the negatively charged FdsB^Glu259^ to either a positively charged or uncharged amino acid led to active enzymes with additional activity with NADP. FdsB^E259R^, FdsB^E259G^, FdsB^E259H^, and FdsB^E259Q^ variants showed specificity toward NADP with a significant decreased affinity with NAD^+^. The *K*_M_ value for NAD^+^ increased up to 62 times compared to the wild-type enzyme. A previous study on the diaphorase subunit of *E. coli* complex I, with a glutamate at a structurally similar position, when replaced with glutamine or histidine, also led to higher affinity toward NADPH [33], showing the importance of this residue in determining the cofactor specificity.

The FdsB^E259D^ variant, on the other hand, had a reduced affinity with NAD^+^ but no affinity with NADP^+^, indicating that the negative charge at this position is important for the binding of NAD^+^ or NADP^+^. Introducing a positive charge (FdsB^E259R/H^) at this position seems to support the hypothesis that a negatively charged 2′-phosphate group of NADP^+^ is bound by a positively charged residue. This is generally true for enzyme engineering studies, where the opposite charge has to be maintained in the enzyme [35].

FdsB^Lys276^ and FdsB^Leu279^ residues are also in the vicinity of NADH. Both the FdsB^K276A^ and FdsB^L279R^ variants had additional activity with NADP^+^. Although both residues do not seem to directly make a hydrogen bond with NADH, they appear to play a role in determining the cofactor specificity. Unfortunately, we were not able to see any additive effects when variants at FdsB^Glu259^, FdsB^Lys276^, and FdsB^Leu279^ were combined, as shown in Table 1. This likely indicates that, in addition to these three residues, other residues are also involved in changing the complete specificity from NAD^+^ to NADP^+^ in order to accommodate the phosphate binding site.

The generated variants were further tested for their CO_2_ reduction activity using NADPH as the reductant and phosphite dehydrogenase as the NADPH-recycling enzyme, as shown in Figure 4a. Similar recycling systems for metal-containing formate dehydrogenase from *Methanococcus vannielii* [17,18] and *Methylobacterium extorquens* AM1 [36] have been previously used and have been shown to work with wild-type enzymes. With the RcFDH variants, we were able to turn over 100 µM of NADPH. FdsB^L279R^, FdsB^E259G^, and FdsB^E259R^ variants were able to produce 100 µM or higher amounts of formate overnight while using NADPH as the coenzyme. The steady-state kinetics of the FdsB^E259G^ variant for CO_2_ reduction showed that it can bind the NADPH cofactor with a higher affinity and has substrate inhibition compared to NADH^+^ (with a *K*_i_ of 233 µM).

Overall, our results are promising and open new routes for potential coupling partners for RcFDH in cascade reactions. Novel NADPH-dependent systems can be coupled with the RcFDH variants described in this report; for example, for light-driven CO_2_ reduction, light-dependent NADPH recycling systems can be used [19,37].

## 4. Materials and Methods

### 4.1. Site-Directed Mutagenesis

Site-directed mutagenesis was carried out using the QuikChange lightening kit (Agilent technologies, Santa Clara, CA, USA). *Rc*FDH wild-type (WT) Plasmid pTHfds05 was used as a template for single amino acid exchanges, while in the case of the double mutants, plasmids with a single mutation were used as the template. The manufacturer’s protocol was followed while using 100 ng of template DNA. Base-pair exchanges were confirmed by DNA sequencing (Eurofin Genomics, Ebersberg, Germany).

### 4.2. Protein Purification

*R. capsulatus* FDH was expressed from *E. coli* MC1061 cells containing the plasmids pTHfds05 and pTHfds07 described in a previous study [21] and purified according to the published procedure. A similar procedure was used for the expression and purification of the RcFDH variants. A total of 75 mM Kpi buffer containing 10 mM sodium azide, pH 7.5, was used as storage buffer. A 75mM KP_i_ buffer, pH 7.5, was used as the assay buffer, and PD-10 desalting columns (Sephadex G-25 M; Cytiva Life Siences, Marlborough, MA, USA) were used for the buffer exchange.

### 4.3. Enzymatic Assays

Enzyme activities were measured aerobically using a UV-2401 PC spectrophotometer (Shimadzu Europa, Duisburg, Germany) at 30 °C. Absorption of NADH/NADPH at 340 nm (ε_NADH/NADPH_ = 6.22 mM^−1^cm^−1^) was followed to determine the rate of formate oxidation. For formate oxidation activities, the reaction mixture contained 5 mM sodium formate and varying concentrations of NAD^+^ between 0.05 and 20 mM. In the case of NADP^+^, concentrations between 0.5 and 20 mM were used. The reactions were started by adding 10 µL of enzyme to a final concentration of 10–200 nM, depending on the variant used. All the measurements were performed in 100 mM Tris-HCl buffer, pH 9.0. Sodium azide, with a final concentration of 50 µM, was present in the reaction mixture.

For CO_2_ reduction kinetics, the reaction mixture contained 100 mM potassium bicarbonate and varying concentrations of either NADH or NADPH (0.005–0.5mM). The volume was made up to 490 µL using 100 mM KP_i_, pH 6.8. The reaction was started by adding 10 µL of enzyme to a final concentration of 0.5–1 µM based on the variant. Kinetic data were processed to obtain the Michaelis–Menten parameters reported herein using OriginPro 2021 (OriginLab Corporation; Northampton, MA, USA).

### 4.4. CO_2_ Reduction Reactions

CO_2_ reduction using formate dehydrogenase was carried out in 2 mL glass vials under semi-anaerobic conditions. A total of 500 µL of reaction volume contained 100 mM of sodium bicarbonate, 100 µM of NADH or NADPH, 10 mM sodium phosphite, 4 µM of phosphite dehydrogenase (PTDH), 1 µM of FMN and 50 mM of KP_i_ buffer, pH 6.8. The reaction was started by adding 2–10 µM of RcFDH^WT^ or variants, and the vials were incubated for up to 18 h at room temperature. The reaction was stopped by adding 30 µL of acetone to 100 µL samples, followed by centrifugation at 14,000× *g* for 10 min. The supernatant was collected for formate detection.

### 4.5. Determination of Cofactor Saturation

Iron, molybdenum, and iron contents were determined using Inductively Coupled Plasma Optical Emission Spectroscopy (ICP-OES). A total of 500 µL of 10 µM enzyme was wet-washed at 100 °C overnight with an equivalent volume of 65% HNO_3_. These samples were diluted by adding 4 mL of deionized water and applied to Optima 2100 DV instrument (PerkinElmerLife Sciences, Waltham, MA, USA). Multielement standard XVI was used for quantification and caliberation. Calculations were based on the assumption that 1 protomer of RcFDH enzyme, when fully loaded with cofactors, contained 1 atom of molybdenum and 24 atoms of iron. The previously published protocol [21] was followed for the quantification of FMN.

### 4.6. Formate Detection Using Gas Chromatography-Mass Spectrometry (GC-MS)

Formate derivatization was carried out using the derivatizing agent, 2,3,4,5,6-pentafluorobenzyl bromide (PFBBr) following adaptation of a previously published protocol [23]. A total of 100 µL of standard/enzyme-free samples was mixed with 50 µL of 325 mM phosphate buffer, pH 8.5. A volume of 365 µL of 100 mM PFBBr solution, which was prepared in acetone, was added to this. Then, the resulting solution was incubated at 60 °C for 20 min after mixing for 1 min using a vortex mixer. After incubation, once the mixture reached room temperature, 500 µL of n-hexane was added and mixed again for 1 min using vortex mixer. Phases were separated by centrifuging at 13,000 rpm for 1 min, and the upper phase was transferred to 2 mL GC vials. The samples were analyzed using GC-MS QP2010 SE instrument (Shimadzu Europa; Duisburg, Germany) containing DB-WAX UI column (30 m × 0.32 mm × 0.25 µm, Agilent). The twelve-minute-long temperature program consisted of an initial temperature of 50 °C for 2 min, followed by heating the column to 220 °C at a rate of 30 °C per minute, and the column was held for three mins at this temperature before cooling down to 50 °C in the next 2 min. The MS interface and the ion source temperatures were kept at 260 °C and 220 °C, respectively. Selected ion monitoring (SIM) mode was used for the detection of derivatized formate. The derivatized product, 2,3,4,5,6-pentafluorobenzyl formate, had a retention time of 5.75 min. In order to determine formate concentration, a calibration curve was generated measuring distinct amounts of formate in buffer.

## Figures and Tables

**Table 1:** Kinetic parameters for formate oxidation of RcFDH^WT^ and variants using NAD^+^ and NADP^+^ as electron acceptors.

	NADP^+^			NAD^+^		
	*K*_M_ ^a^ (mM)	*k*_cat_ ^a^ (min^−1^)	*k*_cat_/*K*_M_ (min^−1^ mM^−1^)	*K*_M_ (Mm)	*k*_cat_ (min^−1^)	*k*_cat_/*K*_M_ (min^−1^ mM^−1^)
RcFDH^WT^	ND ^b^	ND		0.12 ± 0.001	3565 ± 105	29,708
FdsB^E259R^	3.31 ± 1.4	367 ± 45	105.7	7.48 ± 2.1	863.6 ± 56	115.06
FdsB^E259K^	5.2 ± 1.0	342 ± 40	68.4	4.36 ± 1.8	354 ± 55	81.2
FdsB^E259G^	5.5 ± 0.8	551 ± 78	112	3.0 ± 1.2	551 ± 95	183.7
FdsB^E259Q^	6.3 ± 0.7	496 ± 70	78.7	3.34 ± 1.7	1508 ± 120	451.5
FdsB^E259D^	ND	ND		2.75 ± 0.8	1545 ± 140	515
FdsB^K157R^	ND	ND		ND	ND	
FdsB^K157S^	ND	ND		ND	ND	
FdsB^L279R^	5.4 ± 1.7	405 ± 56	75	6.4 ± 1.7	736 ± 84	115
FdsB^K276A^	4.2 ± 1.0	350 ± 39	83.3	6.8 ± 1.5	943 ± 114	138.6
FdsB^K276A/L279R^	5.2 ± 0.7	480 ± 29	92.3	7.0 ± 2.0	687 ± 78	98.1
FdsB^E259R/L279R^	3.9 ± 0.9	347 ± 42	89	5.9 ± 2.2	766 ± 98	129.8
FdsB^E259G/L279R^	4.1 ± 0.9	381 ± 51	92.9	6.2 ± 1.8	629 ± 74	101.4

^a^ Formate oxidation activities were measured aerobically using a UV-2401 PC spectrophotometer (Shimadzu Europa, Duisburg, Germany) at 30 °C. Absorption of NADH/NADPH at 340 nm (ε_NADH/NADPH_ = 6.22 mM^−1^cm^−1^) was followed to determine the rate of formate oxidation. The reaction mixture contained 5 mM sodium formate and varying concentrations of NAD^+^ between 0.05 and 20 mM. In the case of NADP^+^, the concentrations were between 0.5 and 20 mM. The reactions were started by adding 10 µL of enzyme to a final concentration of 10–200 nM, depending on the variant used. All the measurements were performed in 100 mM Tris-HCl buffer, pH 9.0. Sodium azide with a final concentration of 50 µM was present in the reaction mixture. Kinetic data were processed to obtain Michaelis–Menten parameters using OriginPro 9.1 (OriginLab, Northampton, MA, USA) software. ^b^ ND: not detectable.

## Data Availability

Data are contained within the article.
